# Complement 3 (C3) within the hypothalamic arcuate nucleus is a potential key mediator of the effect of enhanced nutrition on reproductive development in young bull calves

**DOI:** 10.1186/s12864-025-11656-0

**Published:** 2025-05-09

**Authors:** Kate Keogh, Stephen Coen, Pat Lonergan, Sean Fair, David A Kenny

**Affiliations:** 1 Teagasc Animal and Grassland Research and Innovation Centre, Grange, Dunsany, Co. Meath, Ireland; 2 School of Agriculture and Food Science, University College Dublin, Belfield, Dublin 4, Ireland; 3 Laboratory of Animal Reproduction, Department of Biological Sciences, University of Limerick, Limerick, Ireland

**Keywords:** RNAseq, Proteomics, Network analysis, C3, ARC

## Abstract

**Background:**

Reproductive development may be advanced in bull calves through enhanced dietary intake during the early life period. This effect between enhanced nutrition with subsequent earlier reproductive development is orchestrated through signalling within the hypothalamic-pituitary-testicular axis. Within the hypothalamus, the arcuate nucleus (ARC) is crucial for the integration of peripheral metabolic status with subsequent gonadotropin releasing hormone (GnRH) signalling; however, the precise molecular control regulating this effect is not fully known. The aim of this study was to evaluate the global transcriptomic and proteomic responses to varied plane of nutrition during early calf-hood in young dairy bull calves. Additionally, we sought to integrate these ‘omics’ datasets to determine key genes and proteins contributing to earlier reproductive development. Between 2–12 weeks of age, 30 Holstein-Friesian bull calves (mean age: 17.5 days; mean bodyweight 48.8 kg), were offered either a high or moderate plane of nutrition with 15 calves in each group. At 12 weeks of age, all calves were euthanised and the ARC tissue isolated from each calf. The ARC tissue was then used for global transcriptomic (miRNAseq and mRNAseq) and proteomic analyses.

**Results:**

Bioinformatic analyses were undertaken to determine differentially expressed transcripts (FDR < 0.1; fold change > 1.5) between the dietary treatment groups, resulting in the identification of 1 differentially expressed miRNA (miR-2419-3p) and 83 differentially expressed mRNA in the ARC region. mRNA target gene prediction identified Complement 3 (*C3*) as a target of miR-2419-3p, suggesting a relationship between the two transcripts. Furthermore, through a co-regulatory network analysis conducted on the proteomics dataset, C3 was revealed as a hub protein. Additionally, through the proteomic network analysis, C3 was interacting with proteins involved in both insulin and GnRH signalling, highlighting a potential role for C3 in mediated the effect of enhanced nutritional status with earlier reproductive development within the ARC.

**Conclusion:**

This study highlights an effect of altered plane of nutrition in early life on the molecular control of the hypothalamic ARC. Additionally, results generated suggest a potential role for the C3 gene in mediating the interaction between enhanced metabolic status with reproductive development within the ARC, regulated by miR-2419-3p.

**Supplementary Information:**

The online version contains supplementary material available at 10.1186/s12864-025-11656-0.

## Background

Improved early life nutrition in calves can lead to positive effects on calf growth and performance as well as providing positive latent effects for many economically important traits. For instance, overall lifetime growth can be improved [1, 2] as well as subsequent beneficial impacts on carcass composition [3]. Moreover, in addition to the benefits on growth and carcass characteristics, improved nutrition during the first 3 to 4 months of life can also contribute to more precocious reproductive development in both heifers and bulls [4]. In male calves, this effect is exerted through complex biochemical signalling between tissues of the hypothalamic-anterior-pituitary-testicular (HPT) axis [5]. Through this biochemical signalling cascade peripheral tissues throughout the body can convey the metabolic status of the body to the hypothalamus which can lead to production of gonadotropin releasing hormone (GnRH) within the hypothalamus. GnRH secreted from the hypothalamus can in turn signal to the anterior pituitary gland for the production of the gonadotropins; luteinising hormone (LH) and follicle stimulating hormone (FSH), the gonadotropins then signal to the testes leading to the production of testosterone and spermatogenesis, respectively. Of the tissues of the HPT signalling pathway, the hypothalamus is crucially important due to its function in overall body homeostatic regulation [6], with peptides and hormones secreted from peripheral tissues signalling to the hypothalamus, which then elicits subsequent downstream effects. For example, hormones including leptin, ghrelin, and insulin, secreted from peripheral tissues, may signal to the arcuate nucleus (ARC) region of the hypothalamus to convey metabolic status of various tissues, which in turn influences the regulation of the hypothalamic decapeptide, GnRH, the gatekeeper of mammalian reproductive development and function [4]. Thus, through enhanced dietary intake in early life, more pronounced GnRH secretion may be achieved, resulting in more precocious pubertal onset in young bulls [7, 8].

Enhanced reproductive development and subsequent earlier pubertal onset as a consequence of augmentation of early life nutritional management, is of particular interest in relation to the collection of semen from genetically elite young sires. Within weeks of birth, genetically elite young bulls may be identified through genomic selection processes for possible future use in artificial insemination. However, although these bulls are identified at a very young age, it is not possible to collect semen until they reach puberty at approximately one year of age [9], when demand often far exceeds supply. Work from our own group has highlighted that puberty can be consistently advanced when a higher plane of nutrition is offered to bull calves during the first 6 months of life [10]. This advancement in age at puberty is associated with the earlier occurrence of the transient rise in systemic concentrations of LH which typically occurs between two and five months of age [11]. Following this a cascade of physiological events lead to earlier testicular histological development, steroidogenesis, spermatogenesis and spermiogenesis, Thus, augmenting early life nutritional management advances the age at which saleable semen can be collected from genetically elite bulls thus shortening the generation interval, a major factor governing the speed of genetic progress in cattle breeding [4, 12]. Indeed, previous work conducted by our group [13] and others [14] established clear evidence for a substantial and extensive influence of enhanced dietary intake during the first 16–24 weeks of life on the transcriptome of testes parenchyma. Furthermore, work from our own group highlighted earlier testicular development evidenced by advanced morphological characteristics of the testes of the same bull calves used for our previous transcriptomic evaluation [13, 15]. This included for example, a greater percentage of spermatogonia and number of Sertoli cells in calves that consumed more feed during the first 18 weeks of life compared to a control group offered a standard diet [15]. However, despite clear effects on the testicular transcriptome in that study, fewer differences were apparent within the other tissues of the HPT axis, namely the hypothalamus and the anterior pituitary; indeed, no genes were detected as differentially expressed between dietary treatment groups in the ARC region of the hypothalamus [13]. Thus, results from that study suggest that the pre-pubertal transient rise in systemic concentrations of LH, which typically occurs between 8 and 20 weeks of life, may have occurred prior to 18 weeks of age and that an earlier stage of development may capture the molecular response of enhanced feeding within the hypothalamic and anterior pituitary tissues.

Thus, although it is clear that signalling within the HPT axis and consequent reproductive development may be affected by metabolic status [16], with evidence of enhanced nutrition in early life advancing the age at puberty in bull calves [4], the precise molecular control regulating this effect is not fully known. Therefore, the aim of this study was to evaluate the global transcriptomic and proteomic responses in the hypothalamic ARC to varied plane of nutrition during the first 12 weeks of life in bull calves. Both messengerRNA (mRNA) and microRNA (miRNA) profiles were assessed in an effort to understand alterations in the expression of protein coding genes as well as the potential contribution of miRNA regulatory processes as a consequence of prevailing dietary intake. A secondary objective was the further integration of transcriptomic and proteomic data through co-regulatory network analyses, in order to identify key regulatory genes, which may hold potential for use as candidates in genomic selection processes.

## Methods

### Animal model and tissue collection

The animal model utilised in this study was originally undertaken to examine the effect of either a high or moderate dietary intake during the early post-natal period on reproductive development in the bull calf and is described in full in Coen et al. [17]. Briefly at 2 weeks of age, 30 Holstein-Friesian bull calves were stratified into two groups balanced for age, bodyweight, farm of origin (4 separate farms) and sire (17 sires in total) and evenly divided into the two dietary treatment groups: high (HI; *n* = 15) or moderate (MOD; *n* = 15) plane of nutrition. From 2 to 12 weeks of age, calves received their respective dietary allowance designed to support growth rates > 1 kg/day for HI and 0.50 kg/day for MOD. All calves were weighed at the beginning and end of the trial and also at weekly intervals. Following 10 weeks of differential feeding (12 weeks of age) all calves were euthanized using a lethal dose of sodium pentobarbitone (300 mg/mL: 0.25 mL/kg bodyweight) administered intravenously [17]. The ARC of the hypothalamus was separated from the remainder of the hypothalamus gland as previously described in Komatsu et al. [18]. All instruments used for tissue collection were sterilised prior to use. Tissue samples were washed in Dulbecco’s phosphate buffered saline (DPBS), snap frozen in liquid nitrogen and subsequently stored at -80° C.

### miRNA- and mRNA-sequencing

A detailed description of RNA isolation, RNA-sequencing and bioinformatic analyses is provided in Coen et al. [19]. Briefly, the Qiagen RNeasy Plus Universal Mini kit was used to separate total RNA from all ARC tissue samples (HI: *n* = 15; MOD: *n* = 15) according to the manufacturer’s instructions including adherence to appendix C for the isolation of small RNAs. Due to the low amount of available tissue within the ARC region, an average of approximately 20 mg of tissue was used to isolate RNA. Yield of isolated RNA was determined by measuring the absorbance at 260 nm with a Nanodrop spectrophotometer and RNA quality was determined through evaluation of the RNA integrity number (RIN) using the RNA 6000 Nano LabChip kit on an Agilent 2100 Bioanalyser, ensuring a minimum RIN value of 8 across all samples. Library preparation and sequencing for both mRNA and miRNA sequencing analyses were undertaken by a commercial service provider (Macrogen Europe Inc., Amsterdam, The Netherlands). Individual cDNA libraries were prepared using the Illumina Truseq stranded mRNA kit (HI: *n* = 15; MOD: *n* = 15) and the Illumina small RNA kit (HI: *n* = 15; MOD: *n* = 15) for mRNA and miRNA analyses, respectively. mRNA-sequencing and miRNA-sequencing were performed on an Illumina NovaSeq (150 bp paired end sequencing) and an Illumina HiSeq 2500 sequencer (50 bp single end sequencing), respectively. Sequencing depth for each analysis was selected based on Illumina guidelines and the ENCODE best practises for RNA sequencing.

Quality of raw sequencing reads was assessed for both mRNA and miRNA datasets using FastQC (version 0.11.8) [20], followed by removal of Illumina sequencing indexing adapters (Cutadapt, version 1.18.8) [21]. Cutadapt was also used to remove short and long reads (shorter than 15 bp, longer than 28 bp [19, 22–24] in order to specifically capture miRNA species from the miRNA sequencing data. Reads retained within the miRNA dataset were subsequently filtered for other species of bovine short RNA (rRNA, tRNA, snRNA, snoRNA, downloaded from https://rnacentral.org/). The miRDeep2 package (version 2.00.8) together with the bovine reference genome (ARS-UCD1.2) and the known bovine mature miRNA sequences and their precursor sequences from the miRBase database [19] was used to profile miRNA expression. Bowtie (version 1.1.1) and STAR (version 2.5.2.b) [25] were then used to align miRNA reads and mRNA reads, respectively to the bovine reference genome (ARS-UCD1.2). Aligned reads were subsequently quantified using miRDeep2 and STAR for miRNA and mRNA datasets respectively. mRNA and miRNA differentially expressed between dietary treatment groups were then determined using edgeR (version 3.26.7). TargetScan (release 7.2; http://www.targetscan.org/vert_72/) was employed to predict mRNA target genes of miRNA identified as differentially expressed between HI and MOD calves.

### Mass spectrometry analysis

A detailed description of the sample preparation and mass spectrometry analysis is outlined in full in Coen et al. [19]. Proteins were extracted from each tissue sample using a tissue homogenizer (TissueLyser II, QIAGEN) and digested using a commercial iST Kit (PreOmics, Germany). A Q Exactive HF-X mass spectrometer was employed for mass spectrometry analysis, followed by MaxQuant (version 1.6.2.3) for processing of raw mass spectrometry data. Protein identification was undertaken using the integrated Andromeda search engine [26]. Differences in protein abundance between HI and MOD calves were determined using the SRMService R package [27].

### Integration of omics results

In order to examine any potential relationships between the transcriptomic and proteomic datasets, an integrative analysis was conducted whereby differentially expressed miRNAs and their target mRNAs which were also differentially expressed were evaluated for their interactions within the proteomics data. Network analysis of the proteomics dataset was undertaken using the weighted gene co-expression network analysis (WGCNA) R software program [28], following Log_2_ transformation of the proteomics data in R. The automatic network construction and module detection method in WGCNA was subsequently used to generate unsigned co-expressed networks as previously described by Coen et al. [19]. Adjacency matrices of proteomics data were then calculated by raising the co-expression matrix to a soft-threshold power of 14 in order to reach scale-free topology of the network (R^2^ > 0.09) for the dataset. The soft-threshold power of 14 was selected as this was the lowest power for which the scale-free topology fit index curve plateaued upon reaching a high value of 0.09. The topology overlap matrix was then calculated, followed by application of average linkage hierarchical clustering to the topology overlap matrix resulting in the grouping of modules of co-expressed proteins [19]. Modules or clusters of co-expressed proteins were then mined for genes of interest based on the relationship between differentially expressed miRNA and predicted target mRNA genes which were also differentially expressed.

### Biological pathways analysis

Ingenuity Pathway Analysis (IPA; Qiagen Inc., https://www.qiagenbioinformatics.com/products/ingenuitypathway-analysis [29] was used to determine biological pathways and functions enriched based on miRNA, mRNA and proteomic analyses of the ARC of bull calves fed either a HI or MOD diet during early life.

## Results

### Animal performance

Details related to animal performance including feed intake and growth as well as physiological characterisation are described in full in Coen et al. [17]. Following completion of the dietary trial at 12 weeks of age, HI calves were 24.7 kg heavier on average compared to MOD calves (*P* < 0.001). High plane of nutrition calves also consumed more milk replacer compared to the MOD calves (*P* < 0.0001). Overall, average daily growth rates were greater for HI calves compared to MOD calves (HI: 0.88 kg/d; MOD: 0.58 kg/d; *P* < 0.001).

### MiRNAseq analysis

On average, 15 million reads were generated per sample following miRNA sequencing, with an associated alignment rate of 92.7%. One miRNA, miR-2419-3p was observed to be differentially expressed between the HI and MOD dietary groups (FDR = 0.049; fold change = 2.14). Predicted mRNA targets of miR-2419-3p, determined through TargetScan analysis are presented in Additional Table 1. Pathway analysis of target genes resulted in enrichment (*P* < 0.05) of pathways including insulin receptor signalling, adipogenesis and both AMPK and sirtuin signalling. Details of all enriched pathways pertaining to mRNA targets of miR-2419-3p are outlined in full in Additional Table 2. Raw sequencing reads from miRNAseq analysis for each sample utilised in this study have been deposited within NCBI’s Gene Expression Omnibus and are available through GEO ID GSE277781.

### mRNAseq analyses

**Fig. 1:**
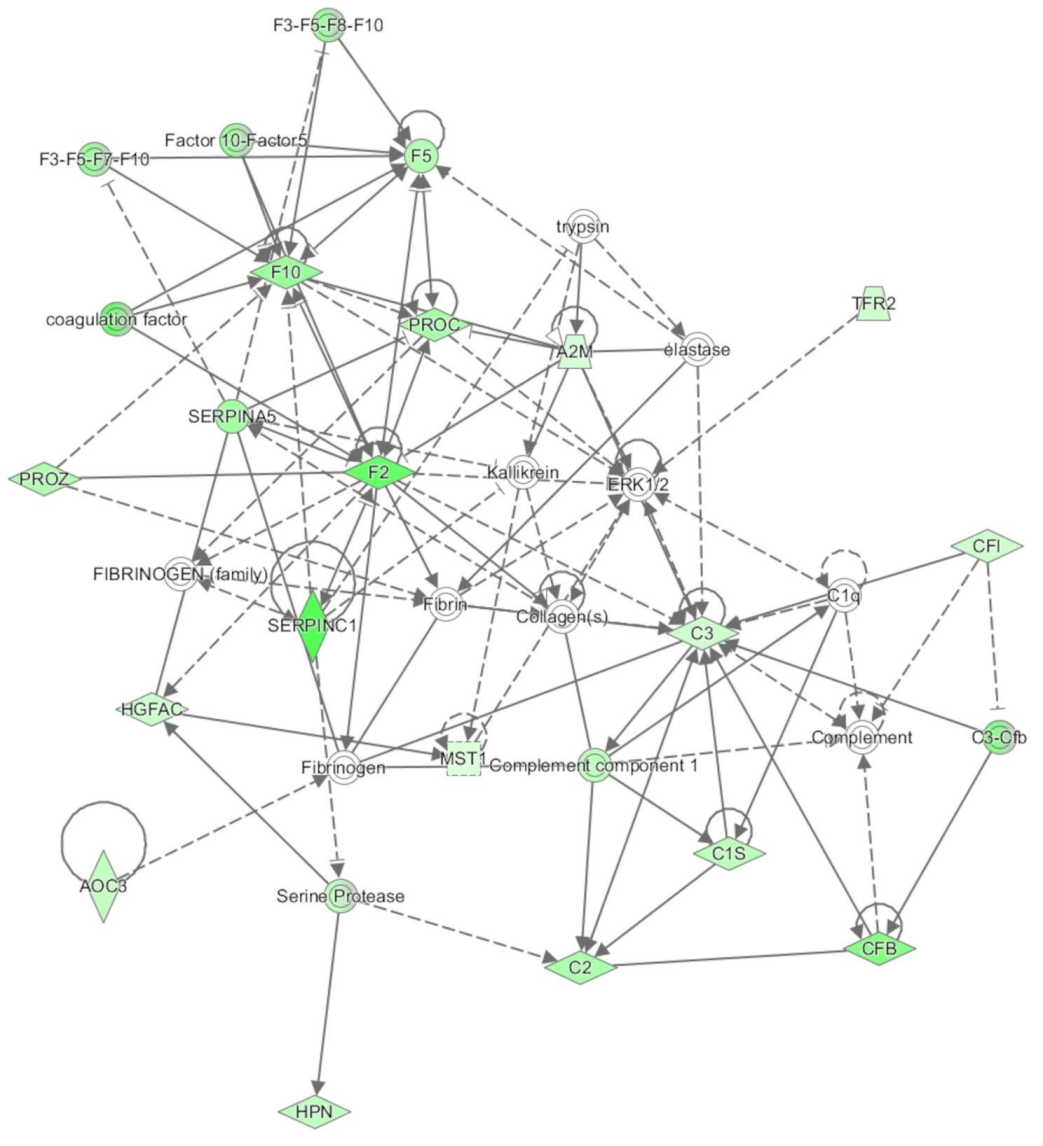
Network involving genes of the complement immune signalling cascade altered in the arcuate nucleus following varied dietary intake of bull calves for the first 12 weeks of life. Network (2) generated through the use of IPA (Qiagen Inc., https://www.qiagenbio-informatics.com/products/ingenuity-pathway-analysis [26]. Genes are displayed as nodes within the network, with green and red nodes representing genes down- and up-regulated in the calves fed the high plane of nutrition compared to those fed a moderate plane of nutrition up to 12 weeks of age. The node colour intensity indicates the magnitude of differential expression

**Fig. 2:**
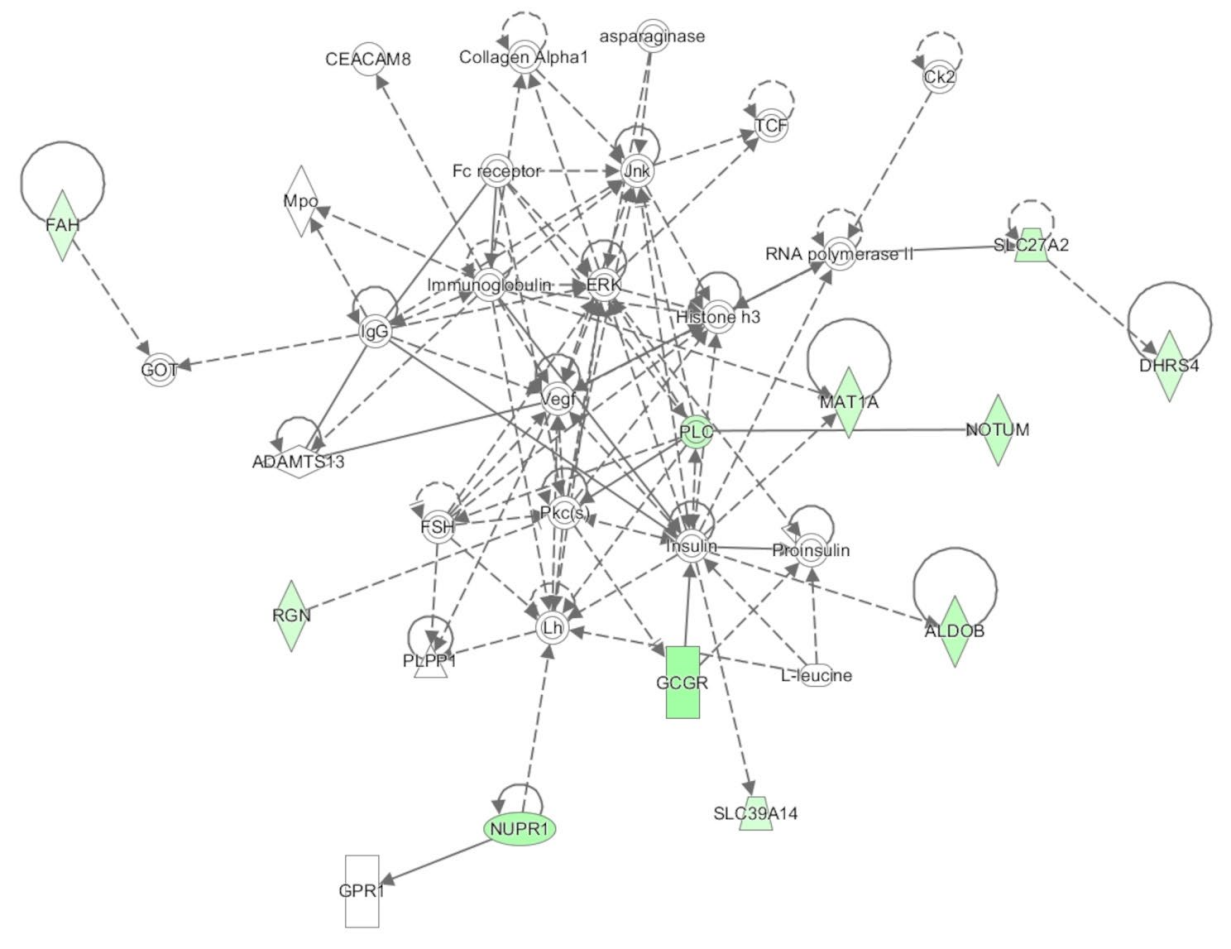
Network involving genes involved in amino acid metabolism and small molecule biochemistry altered in the arcuate nucleus following varied dietary intake of bull calves for the first 12 weeks of life. The network includes differentially expressed genes as well as insulin and gonadotropins, indicating a role for the genes highlighted in green in mediating reproductive development as a consequence of enhanced early-life nutrition. Network (4) generated through the use of IPA (Qiagen Inc., https://www.qiagenbio-informatics.com/products/ingenuity-pathway-analysis [26]. Genes are displayed as nodes within the network, with green and red nodes representing genes down- and up-regulated in the calves fed the high plane of nutrition compared to those fed a moderate plane of nutrition up to 12 weeks of age. The node colour intensity indicates the magnitude of differential expression

Across the mRNA sequencing data an average of 70 million sequencing reads were generated. An average mapping rate of 86.2% was achieved following alignment of trimmed sequencing reads to the bovine genome. Through differential expression analysis between treatment groups within EdgeR, 83 genes were identified as differentially expressed (FDR < 0.1; fold change > 1.5; Additional Table 3). Furthermore, pathway analysis of differentially expressed genes revealed enrichment (*P* < 0.05) of numerous biochemical pathways including those related to immune signalling processes, for example coagulation system, acute phase response signalling and complement system (Additional Table 4). Network analysis undertaken in IPA also revealed two networks of interest -haematological disease, organismal injury and abnormalities (network 2; Fig. 1) and amino acid metabolism and small molecule biochemistry (network 4; Fig. 2). Details of IPA networks are presented in Additional Table 5. Raw sequencing reads for the mRNAseq dataset utilised in this study have been deposited within NCBI’s Gene Expression Omnibus and are available through GEO ID GSE278108.

### Global proteomics analysis

In total 5,569 proteins were identified across the ARC samples, representing those with a maximum of 10 missing values per protein and with at least two peptides. However, despite identifying 5,569 proteins, none were considered significantly differentially abundant between treatment groups following multiple testing correction (FDR < 0.1; fold change > 1.5). The full list of proteins identified in the ARC tissue based on an uncorrected p-value (*p* < 0.05) is presented in Additional Table 6. Of particular interest was the differential abundance of C3, TF and HP, which were also differentially expressed at the mRNA level. Proteomics data generated in this analysis have been uploaded to the ProteomeXchange Consortium via the PRIDE (http://www.ebi.ac.uk/pride) partner repository [30] with the data identifier PXD055939.

### Integrative network analyses

**Fig. 3:**
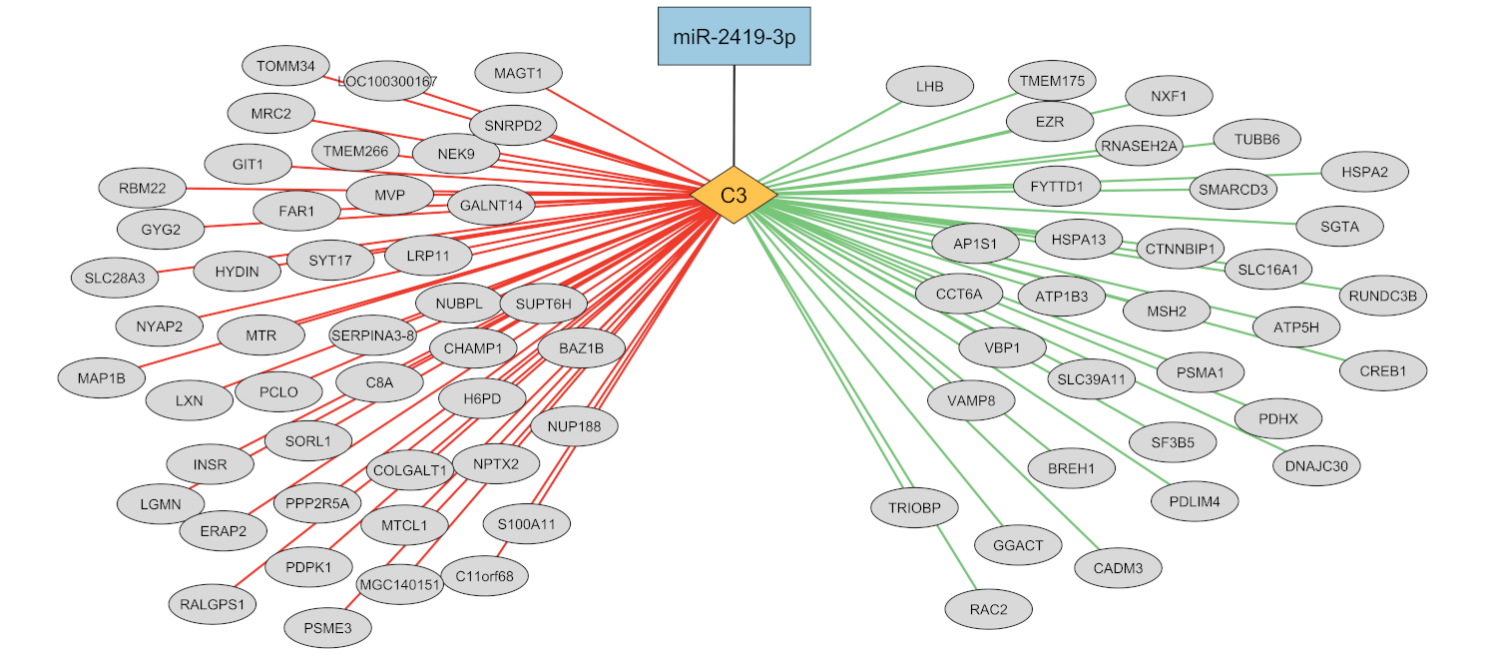
Integration of miRNA, mRNA and protein networks following 12 weeks of altered plane of nutrition in young bull calves. Differential expression of miR-2419-3p and *C3* within the arcuate nucleus transcriptomes, indicated a relationship between these two transcripts, with miR-2419-3p up-regulated and *C3* down-regulated in the HI calves. Co-regulatory network analysis of proteins identified within the arcuate nucleus revealed *C3* as a hub protein, proteins connected to C3 by a red line indicate those regulating C3, whilst green lines indicate proteins being regulated by C3

Co-regulatory network analysis of the proteomics dataset undertaken through WGCNA resulted in the formation of 9 separate networks of proteins. Proteins co-regulated within each separate network are detailed in Additional Table 7. Of the predicted target mRNA genes of miR-2419-3p, only 2 genes, *C2* and *C3*, were identified as differentially expressed between HI and MOD calves at the mRNA level. Additionally whilst miR-2419-3p was up-regulated in the HI calves, both *C2* and *C3* were down-regulated indicating a relationship between these mRNA genes and miRNA. From the co-regulatory networks generated, the C3 protein was found to be interacting with over 70 additional proteins (Fig. 3). The C2 protein was not identified as significantly connected to any other proteins through network analysis.

## Discussion

Enhanced early life nutrition in calves is key to maximising their subsequent lifetime growth potential as well as benefiting the composition of the emanating saleable meat [3, 31]. Furthermore, in addition to improving growth potential, dietary augmentation during the early life period is also known to influence the complex interactions between metabolic status cues and reproductive development, mediated through the HPT signalling axis [7]. In particular, the ARC region of the hypothalamus is centrally involved in receiving and interpreting signals related to metabolic status throughout the body and eliciting subsequent downstream effects [32, 33]. Indeed, results from this study showed a clear effect of differential plane of nutrition during early life on the miRNA and mRNA transcriptomes of the ARC. This is in contrast to an earlier study which failed to detect transcriptional differences within the ARC in response to increased dietary intake in bull calves at 18 weeks of age [13]. This may suggest that acute neuroendocrine effects of enhanced nutrition and metabolic status during early life on gonadotropin secretion were already well established prior to 18 weeks of age. However, despite identifying differences in both mRNA and miRNA transcript abundance between HI and MOD calves, the same outcome was not apparent through the proteomics analysis, where no protein was identified as significantly differentially abundant following correction for multiple testing. This lack of significant difference at the protein level may be attributable to variation in methodologies employed between transcriptomic and proteomic analyses [34], as well as limitations in the dynamic range of proteins detected which may have resulted in lower abundance proteins not being sufficiently detected and the potential impact of post-translation modifications on overall protein abundance detection.

Global miRNA analysis in the current study resulted in the identification of one differentially expressed miRNA, miR-2419-3p, which was up-regulated in the HI calves, with no other miRNA differentially expressed between dietary groups. Differential expression of this miRNA has previously been reported in other bovine tissues. For example, a role for miR-2419-3p in relation to varying plane of nutrition was previously established by Wang et al. [35] apparent through differential expression in the rumen of cattle that were fed varying amounts of low quality forage. Moreover, Billa et al. [36] reported differential expression of miR-2419-3p in the mammary gland of lactating Holstein and Montbeliarde cows. Whilst other studies have identified associations between single nucleotide polymorphisms (SNPs) within the miR-2419-3p gene with milk production traits [37] and fatty acid traits in muscle [38]. The diverse range of functions affected by this miRNA are also evidenced by the large number of mRNA targets as well as their biological functions. For example, from the list of statistically significantly enriched biochemical pathways of miR-2419-3p target mRNAs (Additional Table 2), it is evident that various different biological processes may be affected by this particular miRNA. These include those related to immune signalling processes, signal transduction and intracellular signalling, nervous system development, growth and proliferation and biological pathways related to metabolic status. Additionally, biochemical pathways directly related to dietary plane of nutrition and reproductive processes were also enriched. These included insulin receptor, AMPK and sirtuin signalling pathways as well as adipogenesis which have all been previously implicated in studies with growing cattle where differential feeding regimens were employed [8, 39–41]. Additionally pathways related to reproductive signalling and feeding behaviour including GnRH, opioid and semaphorin neuronal repulsive signalling pathways were also enriched between HI and MOD calves in the current study [8]. Specifically both opioid and semaphorin signalling are involved in feeding behaviour [42] whilst GnRH signalling is involved in GnRH neuronal migration [43], however all have been implicated towards the intersection between enhanced dietary intake in early life with consequent advanced reproductive development [7, 8, 44]. Other miR-2419-3p mRNA targets of relevance included *THRA*, which encodes a thyroid hormone receptor, which may mediate metabolism as a consequence of altered nutrition and the adiponectin receptor (*ADIPOR2*), which mediates energy homeostasis. Moreover, adiponectin secretion may influence gonadotropin release [45]; thus, miR-2419-3p may contribute to reproductive processes through targeting the *ADIPOR2* gene. Additionally, genes encoding activin and inhibin (*ACVR2B* and *INHBA*) were also predicted as mRNA targets of miR-2419-3p. Activin and inhibin function to stimulate the release and inhibit the secretion of follicle stimulating hormone (FSH), respectively, thus, the identification of these genes as targets of miR-2419-3p further emphasises the role of this miRNA in reproductive processes within the ARC region of the hypothalamus. Taken together, these results highlight the diverse range of biological effects invoked by miR-2419-3p whilst also establishing a role for this miRNA in relation to altered metabolism and subsequent reproductive processes in bull calves as a consequence of enhanced nutrition during the early post-natal period.

Differential expression analysis of protein coding genes between calves in the HI and MOD groups resulted in the identification of genes with diverse functions including molecule and nutrient transport as well as genes involved in the immune system. Similarly in other studies examining varying plane of nutrition, genes involved in nutrient or cellular molecule transport were also differentially expressed [13, 14, 41, 46]. In these studies, cellular transporter genes were up-regulated in animals consuming more feed, which may be due to a greater requirement to process nutrients from the diet within the cellular infrastructure [46]. However, in contrast to those studies, results from the current study highlighted reduced expression of transporter related genes in the ARC of HI calves at 12 weeks of age. These included genes encoding members of the ATP-binding cassette transporters, *ABCA10* and *ABCB4*, which are involved in molecular transport across both extra- and intra-cellular membranes, the apopiloprotein lipid transporter, *APOM*; and ceruloplasmin (*CP*), which functions in iron transport across cell membranes. Additionally, genes encoding solute like carrier proteins were also differentially expressed including the cation transporter, *SLC22A18*; fatty acid and amino acid transporters (*SLC27A2* and *SLC38A4*) and the metal and zinc transporters, *SLC39A14* and *SLC39A5*. Additionally, down-regulation of genes involved in both glycolysis and insulin signalling was also apparent in the HI calves, despite the documented higher systemic concentrations of both insulin and glucose in these calves [17]. These genes included *ALDOB*, involved in glycolysis and gluconeogenesis; *DPP4*, involved in glucose and insulin metabolic processes and the glucagon receptor, *GCGR*, which has important functions in controlling glucose homeostasis, as well as the aforementioned *SLC39A14* gene which is also involved in regulating insulin receptor signalling and glucose uptake. Indeed, the relationship between the differentially expressed genes observed here and insulin concentrations is further highlighted through network 4 (Fig. 2), where differentially expressed protein coding genes were included within a network involving insulin. Interestingly, the same network also includes the gonadotropins, FSH and LH, which may indicate towards a relationship between the differentially expressed genes in this network intervening between metabolic status and reproductive development processes. Overall though, results suggest less nutrient processing in the calves which were consuming more feed which may be unexpected given their increased dietary intake in comparison to the MOD calves [17]. However, an alternative perspective may be that the tissue targeted in this study may have already developed within the HI calves and other tissues within the HI calves were then prioritised for growth as opposed to brain tissue at twelve weeks of age compared to the MOD control group.

Further evidence for the ARC tissue to be more developed in the HI calves is provided through the differential expression of genes involved in immune processes, which were all down-regulated in the HI calves. Although these results might indicate an immune response within the MOD calves at the time of sampling, immune cells and associated signalling processes are key mediators of post-natal brain and neural development [47] and their down-regulation in the HI calves may suggest that these calves had developed earlier due to enhanced nutrition in early life. Indeed, in a similar study employing heifer calves differentially fed up to 22 weeks of age, one such gene *HP*, involved in inflammation was also up-regulated in the hypothalamus of calves fed a moderate dietary intake compared to an enhanced intake during the first 22 weeks of life [48]. The effect of immune cells and signalling pathways towards development of the tissue used is further evident through network 2 (Fig. 1), which involved a number of genes involved in the complement cascade including *C1R*,* C1S*,* C2*,* C3*,* CFB*,* CGI*, all of which were down-regulated in HI calves. The complement cascade forms part of the immune system responsible for enhancing the capacity for the removal of microbes and damaged cells from an organism, promoting inflammation and damaging the pathogen’s cell membrane [49]. Components of the complement system have been shown to contribute to neurobiological processes related to brain development including neurogenesis and neuronal migration [50]. Moreover, the importance of the C3 component of the complement cascade has been demonstrated in mouse embryos deficient in C3, which displayed increased proliferation of particular regions of the brain, indicating that components of the complement system inhibit neural progenitor cell proliferation at early stages of development [50]. Thus, an effect of early life plane of nutrition on the complement system within the ARC may suggest differential progression of brain development during the early post-natal period as a direct consequence of dietary intake [8]. Similarly, we also observed an effect of genes involved in fatty acid metabolism, all of which were down-regulated in the HI calves. Due to the function of lipid molecules as key components of the structure and function of the brain, differential expression of these genes may also indicate towards earlier tissue development within the HI calves. Specifically, these genes included the apolipoproteins, *ACOX2* and *APOM* which are involved in lipoprotein metabolism with *ACOX2* involved in the degradation of long branched fatty acids, and the fatty acid desaturase gene, *FADS6*. In a similar study examining early life nutrition up to 18 weeks of age conducted by our group, differential expression of *ACOX2* was also apparent in subcutaneous adipose tissue of bull calves [41]. Furthermore, genes associated with brain tissue development were also identified as differentially affected in the current study, with all of these genes displaying greater expression in the MOD group of calves. These genes included *AGMO*, *AHSG*, *GATM*, and *NOTUM*. *AGMO* encodes an enzyme that cleaves the ether bond of ether lipids, which are essential components of brain development. *AHSG* is involved in brain development and through its function as a key regulatory protein of the Wnt signalling pathway the *NOTUM* gene also contributes to vertebrate brain development [51]. Finally, *GATM* encodes a mitochondrial enzyme that belongs to the amidinotransferase family of enzymes and functions in central nervous system development including the brain. The *GATM* gene was also differentially expressed in the early life nutrition studies of English et al. [13] and Johnson et al. [14] through differential expression within the testes parenchyma in each study. Overall, these results suggest an earlier development of brain tissue, and specifically the ARC region as a consequence of an enhanced nutrition up to 12 weeks of age. The earlier maturation of the ARC region in HI calves may subsequently allow for earlier reproductive processes and signalling through the HPT signalling axis.

In addition to our primary focus of identifying genes and proteins differentially expressed between HI and MOD dietary groups, a secondary objective was to link these varying results to determine the interaction between miRNA, mRNA and proteins. Despite the large number of mRNA genes targeted by miR-2419-3p, only two such genes (*C2* and *C3*) were differentially expressed between HI and MOD calves in the current study. Both *C2* and *C3* displayed lower expression in the HI calves, suggesting an association with expression levels of miR-2419-3p, which was up-regulated in HI calves within the same tissue. As mentioned above, these two genes function in the immune signalling complement cascade and their down-regulation may be due to earlier tissue development in the HI calves. Following correction for multiple testing no proteins were identified as differentially abundant between treatment groups, however C3 abundance was lower in the HI calves, based on the uncorrected p-value. Moreover through network analysis of the proteomics dataset we were able to determine the interaction amongst proteins within the ARC tissue in bull calves at 12 weeks of age. A total of 9 separate networks of co-regulated proteins were derived from the proteomics dataset. Of particular interest was the network which contained the complement protein C3, which as mentioned above we also observed a relationship between miR-2419-3p and *C3* through RNAseq analyses. Similar to the target genes of the mir-2419-3p miRNA, genes contained within the same network as C3 were involved in diverse functions including mTOR signalling, spliceosomal cycle, sirtuin signalling pathway, actin cytoskeleton signalling, synaptogenesis signalling pathway, insulin receptor signalling and GnRH signalling. Furthermore through network analysis, the interactions of C3 with other proteins within the ARC tissue were apparent, which may have been directly affected by miR-2419-3p expression. Interestingly, proteins directly connected to and interacting with C3 included genes with functions related to metabolism as well as proteins involved in reproductive processes. For example, proteins involved in insulin signalling were regulating the abundance of C3, including the insulin receptor, INSR, and PDPK1, whilst the C3 protein was regulating additional proteins involved in GnRH signalling including LHB and CREB1. INSR is the protein receptor for insulin, whilst PDPK1 is also involved in insulin signalling through its role as a serine-threonine kinase by phosphorylating and activating downstream kinases upon ligand-receptor binding [52]. Thus, these results suggest a potential effect of insulin on C3 protein abundance within the ARC. Indeed insulin concentrations were greater in the HI MOD calves [17]. Furthermore, proteins regulated by C3 included the gonadotropin protein LHB as well as CREB1, which induces transcription in response to stimulation from hormone and neuropeptides including somatostatin, TAC1, which is involved in GnRH secretion and NPY [53, 54]. Together these results suggest a potential interaction between insulin receptor and C3, with C3 possibly mediating the effect of insulin signalling to subsequent gonadotropin signalling. Moreover, the larger network which C3 was involved in also included proteins involved in dietary intake for example NPY, and AGRP, which were co-expressed together to stimulate food intake, as well as SEMA4D and NRP1 proteins which are involved in GnRH neuronal migration [43, 55]. Indeed, differential expression of the NPY and AGRP genes was recently observed in a contemporary study in the ARC of heifer calves [48]. These results indicate towards a potential role for the complement protein, C3, towards regulating the effect of enhanced metabolic status, due to increased dietary intake with subsequent earlier reproductive development in bull calves. However additional experiments are required to validate the interaction between mir-2419-3p and C3 as well as the functional relationship between C3 and the aforementioned proteins involved in metabolic and reproductive processes.

## Conclusions

Results from this study highlight an effect of enhanced nutrition during early life on the molecular control of the hypothalamic ARC. Differentially expressed protein-coding genes indicate towards advanced development of the ARC tissue in HI calves in response to their greater nutrient intake during early life, which is consistent with the typically more precocious sexual development observed in such calves. Moreover, through the integration of miRNA and mRNA results, a possible effect of the miR-2419-3P miRNA on *C3* expression was apparent. The potential role of C3 in mediating the response of reproductive development through enhanced nutrition was further evident through an evaluation of the interaction amongst proteins within the ARC apparent through co-regulatory network analysis, highlighting potential interactions between C3 with proteins involved in insulin and GnRH signalling pathways. The results generated in this study broadens our knowledge in relation to potential molecular processes regulating the intersection between metabolic status and reproductive development in the ARC of bull calves during the early post-natal period. However, further functional experiments in independent populations of calves are warranted to confirm the results generated in this study. Furthermore, results from this study provide fundamental insight which may hold potential for the development and utilisation of robust biomarkers which could be harnessed to identify bulls with superior genetic potential for earlier onset of puberty, as well as other economically important traits such as carcass composition, that have been shown to be latently affected by enhanced nutrition during early life.

## Electronic supplementary material

Below is the link to the electronic supplementary material.

Supplementary Material 1

## Data Availability

The sequencing data underlying this article are available in NCBI’s Gene Expression Omnibus at [https://www.ncbi.nlm.nih.gov/geo/] and is publicly available and can be accessed with unique GEO ID [GSE277781] and [GSE278108]. Proteomics data generated in this analysis have been uploaded to the ProteomeXchange Consortium via the PRIDE [http://www.ebi.ac.uk/pride] partner repository [26] with the data identifier: PXD055939.

## Abbreviations

ARC: Arcuate nucleus
DPBS: Dulbecco’s phosphate buffered saline
FDR: False discovery rate
FSH: Follicle stimulating hormone
GnRH: Gonadotropin releasing hormone
HI: High plane of nutrition treatment group
HPT: Hypothalamic-pituitary-testicular axis
IPA: Ingenuity pathway analysis
LH: Luteinising hormone
miRNA: MicroRNA
MOD: Moderate plane of nutrition treatment group
mRNA: Messenger RNA
RIN: RNA integrity number
